# Science communication to the public

**DOI:** 10.3325/cmj.2018.59.43

**Published:** 2018-04

**Authors:** Slobodan Vukičević, Zvonko Kusić, Lovorka Grgurević

**Affiliations:** 1 Laboratory of Mineralized Tissues, Scientific Center of Excellence for Reproductive and Regenerative Medicine, University of Zagreb School of Medicine, Zagreb, Croatia *slobodan.vukicevic@mef.hr*; 2 Croatian Academy of Sciences and Arts, Zagreb, Croatia

We live in a world where scientific issues are subject to endless debates (1) and where “alternative views” are expressed about the influence of science and medicine on the mankind's well-being. Additionally, politicians demonstrate an astonishing negativism and ignorance about issues of immense importance, such as vaccination and global warming (2). In the recent March for Science, people in 600 cities all over the world rallied in the streets to advocate for science and protest against funding cuts and political interference with scientific issues.

A discussion in the *New England Journal of Medicine* prompted us to suggest a somewhat different approach for communicating science from that practiced in the March for Science (2,3). We suggest that it is time to present the fascinating old and new scientific success stories (4), believing that it would reverse disbelief in science among people not familiar with scientific principles.

Political interference with scientific work can be prevented only by ensuring the quality of scientific arguments and the scientists' responsibility for their content. An example of enthusiastic presentation of scientific discoveries is the list of medical breakthroughs published in the *New England Journal of Medicine* upon the journal’s 200th anniversary (5,6). The breakthroughs on this long list, including the first anesthesia, syphilis treatment, discovery of penicillin, first kidney transplantation, link between smoking and lung cancer, introduction of the lung artery catheter, early clinical descriptions of AIDS, and many more (6,7) can indeed be considered world-changing.

When the first surgical ether-inhalation analgesia replaced alcohol-mediated analgesia at the Massachusetts General Hospital in 1846 (8), nobody doubted that it was a medical “miracle.” Similarly, one of the most emotional scenarios in the medical history was the first insulin application performed by Walter Campbell, Almon Fletcher, Frederick Banting, and Charles Best. In the H Ward of the Toronto General Hospital, the doctors injected pancreatic extract (purified by James Bertram Collip) into the thigh of 14-year-old Leonard Thompson. After the initial success, they applied the treatment to other teenage patients. Within hours, the scientists witnessed a “resurrection” of children on the verge of a diabetic coma. This important success story must not only be taught in medical schools but has to reach the general public (9). Bringing children back to life in 1921 and 1922, and thousands of diabetic patients worldwide in the years to come, speaks for itself and has no opponents.

While some scientific “miracles” can be witnessed immediately, it can take years before others produce visible changes. For example, poliomyelitis was eradicated as many as 24 years after Jonas Salk had discovered the cure in 1955. It was a desperate race in a country that feared polio almost as much as it feared nuclear war. The most demanding clinical study in the history of medicine included 20 000 physicians and medical staff, 64 000 school employees, 220 000 volunteers, and more than 1 800 000 children (10). When the news of the successful vaccination broke, the nation celebrated Salk as a “miracle worker,” and April 12, 1955 was almost pronounced a national holiday. Salk rejected to patent the vaccine and said: “There is no patent. Could you patent the Sun?” (11), making the vaccine available to the world. Those were the moments when empathy for patients prevailed against personal and pharmaceutical greediness. Today, polio exists only in Pakistan, but might return to areas with low vaccination coverage.

Ever since 1787, when Jenner first applied cowpox material to protect humans against the smallpox, vaccination has extended peoples' lifespan for many years (12). However, we have recently witnessed dire consequences of anti-vaccination movement in Serbia, Sweden, and Slovenia, where measles epidemic has killed several children whose parents opposed vaccination. Therefore, repeating the history of medicine is not a good avenue to follow (13). Who can take responsibility for the return of deadly diseases removed from the list of medical threats because of unsubstantiated claims that vaccination is associated with autism, diabetes, and immunological diseases (14-16)? Especially when we know that several requirements have to be fulfilled before an association is claimed causative (17).

Numerous scientific discoveries have made people believe in science and turned the United States of America (USA) into the global scientific leader, while the country’s distinguished institutions attracted the world's best scientists. Former US presidents and the Congress did not take sides in scientific discussions but glorified the US scientific contributions, knowing that it will enhance the USA image and maintain the country’s technology domination in the world.

In an attempt to place Europe among the global scientific leaders, the European Parliament founded the European Research Council to finance excellent scientists. This has in the first ten years resulted in six Nobel Prizes, four Fields Medals, five Wolf Prizes, 100 000 articles, 180 researchers moving to Europe, and over 800 issued patents (18).

When presenting scientific results to the public, less publicity should be given to spectacular findings in animal models before they are confirmed in clinical studies. This was the case with anti-angiogenic therapies that erased all types of tumors in mice but were shortly after proven ineffective in humans (19,20). Also, controversies should be avoided in public scientific debates since, as Rosenbaum said (2), when non-political issues become political, the politicians might take an anti-scientific position and the voters will likely follow suit. In such a scenario, polarization becomes unavoidable, diminishing the power of science and potentially leading to a deleterious outcome. In a polarized society, according to Rosenbaum (3), we need to resist human nature, ie, “the impulse to believe what we want to believe.” This way science will continue to receive funding and scientists will have more freedom to explore time-consuming phenomena not immediately attractive to the public.

The March for Science did not have a measurable effect because its political connotations made scientific issues less prominent. Science should speak for itself via its great results rather than via general resistance to its suppression. However, when one treats an incurable group of patients and they continue to live, this is a language that everyone understands, and this gets full public support. We propose to reverse the current disbelief in science by changing the narrative of non-controversial recent and historical scientific contributions to human well-being, demonstrated as science that successfully addresses unmet medical needs.
